# Detecting Patterns of Intimate Partner Violence Using Qualitative Analyses and Machine Learning Algorithms

**DOI:** 10.1007/s11121-026-01923-1

**Published:** 2026-05-06

**Authors:** Ying Zhang, Jun Fang, Ambika Krishnakumar

**Affiliations:** 1 Department of Psychology, Clarkson University, Potsdam, NY USA; 2 Department of Human Development and Family Science, Syracuse University, Syracuse, NY USA; 3 Department of Psychology, Syracuse University, Syracuse, NY USA; 4 Department of Psychological and Brain Sciences, Indiana University, Bloomington, IN USA

**Keywords:** Intimate partner violence (IPV), Machine learning (ML), Text mining, Social media analysis

## Abstract

Intimate partner violence (IPV) survivors increasingly use social media platforms to share their experiences and to seek help and support for their IPV-related concerns. IPV evidence extracted from social media platforms can provide valuable information and complement data obtained from conventional data sources (e.g., self-reports and interviews) thereby enhancing our understanding of IPV victimization. This study addressed three research questions: (1) What range of IPV behaviors emerge through qualitative coding? (2) To what extent do machine learning (ML) based text classifications yield results comparable to qualitative coding of IPV behaviors? and (3) Do the conceptualizations that emerge from unsupervised ML capture additional behaviors or contextual information not identified through qualitative analyses? We analyzed 400 posts from women on IPV-related online forums using qualitative content analysis and two ML approaches: supervised text classification and unsupervised topic modeling (Latent Dirichlet Allocation). Supervised learning approaches, notably Random Forest and Neural Networks, proved effective in classifying IPV violence subtypes with high accuracy (*F*1 scores .62 – .85). A comparison of findings from the qualitative and topic modeling approaches supported the presence of distinct characteristics of IPV: physical and sexual violence, psychological/emotional abuse, and coercive control. The ML model revealed vocabulary patterns consistent with relational and child-related contexts, temporal and frequency indicators of violence, references to legal system engagement, and spatial contexts, elements that were less captured through thematic qualitative coding alone. The consistency of findings across qualitative and ML approaches points to the potential of leveraging ML techniques when analyzing qualitative data, thus enabling the development of timely and effective IPV interventions.

## Introduction

Intimate partner violence (IPV) is a serious public health problem that constitutes a violation of fundamental human rights and threatens the health and well-being of individuals, families, and communities (UN General Assembly, 1993). Violence by an intimate partner or spouse in marital, dating, cohabiting, or other romantic relationships can include a range of abusive acts such as physical violence, sexual coercion, psychological abuse, and coercive controlling behaviors (Krug et al., 2002). Globally, 1 in 3 women between the ages of 15–49 years report physical and/or sexual violence from a current or former intimate partner (Sardinha et al., 2022). Although IPV is an issue of grave concern that disproportionately impacts women, its negative effects cut across race, age, socioeconomic, gender, sexual identity, and relationship status (Renner & Whitney, 2010). Despite the health, social, economic, and quality of life costs for women, many women victims underreport, misreport, or deny their experiences of violence to service providers, medical professionals, and law enforcement officers for fear of reprisal, shame, fear, stigma, and/or because of an inability to identify their experiences as violence (Boethius & Åkerström, 2020; Overstreet & Quinn, 2013).

IPV researchers have mostly depended on information from surveys, court records, shelters, medical records, and police reports to document the prevalence and characteristics of IPV (Ellsberg et al., 2001). Data from these sources have mostly been analyzed using qualitative and quantitative approaches which have been used by researchers to analyze IPV information (Li et al., 2022; Sardinha et al., 2022; Testa et al., 2011). Although qualitative methods are particularly informative and provide rich information, the exhaustive aspects of qualitative data collection (e.g., focus groups, open-ended survey responses, interview transcripts, observations) along with other extensive research procedures (e.g., transcribing, reading extensive and complex textual data, developing meaningful codes and themes, assessing inter-rater reliability, and writing in-depth descriptive reports) have proven to be labor-intensive for researchers (Sutton & Simons, 2015; Thomas et al., 2014).

In recent years, with the advent of social media (e.g., Facebook, Twitter, Reddit, etc.), researchers have access to the narratives posted by IPV survivors about their experiences of violence (Guo et al., 2023). In this paper, we intend to use narrative data from social media posts by IPV survivors to first conduct qualitative analysis followed by the use of supervised and unsupervised ML algorithms (Arias & Fabian, 2021; Chiong et al., 2021) (Supervised Text Classification Process) to access and process IPV narratives. A “text mining” approach that utilizes natural language processing techniques to analyze the semantics, grammatical, linguistic, and syntactic patterns of transcripts will be used to prepare the data for analysis (Allahyari et al., 2017). Next, after identifying the functioning of the best-performing classifier using the supervised text classification process, unsupervised ML (Latent Dirichlet Allocation (LDA) algorithm, i.e., topic modeling) will be used to automate the classification process. Finally, findings from both approaches (qualitative and unsupervised ML) will be compared to assess the potential of machine learning approaches to analyze narratives from social media posts.

### Literature Review

The conceptualization of IPV used in this study was derived from the extant IPV research literature and includes the definition outlined by the World Health Organization (WHO), which characterizes physical violence as encompassing behaviors like “slapping, hitting, kicking, and beating,” sexual violence that includes behaviors such as “forced sexual intercourse and other forms of sexual coercion,” and psychological/emotional violence that includes actions such as “insults, belittling, constant humiliation, intimidation (e.g., destroying things), threats of harm, and threats to take away children” (World Health Organization, 2012). We were also informed by various measures such as the Revised Conflict Tactics Scale (CTS2) (Straus et al., 1996) to assess IPV. In the CTS2, psychological aggression was assessed using eight items (4 minor and 4 severe) that examined verbal and nonverbal aggressive behaviors directed by the perpetrator toward the victim (e.g., insulted or swore at partner, shouted at partner, stomped out of the room, threatened to hit or throw something at partner, and destroyed something of partner). Coercive controlling behaviors are an important characteristic of IPV and are comprised of behaviors such as “isolating a person from family and friends, monitoring their movements, and restricting access to financial resources, employment, education or medical care” (World Health Organization, 2012). Incidents and patterns of “coercive controlling violence” also include perpetrators’ use of power and domineering tactics such as intimidation, isolation, coercion and threats, emotional abuse, minimizing, denying, and blaming, use of children, economic control against the victim (Day & Bowen, 2015). Exerting coercive control in relationships is entrenched in male privilege and male dominance (Myhill, 2015) and is indicative of gender inequality and discrimination faced by women (World Health Organization, 2012). Our conceptualization of coercive control was also informed by The Controlling Behaviors Scale-Revised (CBS-R) (Graham-Kevan & Archer, 2005) that assessed six subscales: economic control (4 items) (e.g., refuse to share money/pay fair share, control the other’s money), threatening control (4 items) (e.g., threaten to harm the other one, threaten to leave the relationship), intimidating control (5 items) (e.g., try to make the other do things he or she did not want to, smash the other one’s property when annoyed/angry, vent anger on pets), emotional control (5 items) (e.g., try to put the other down when getting “too big for his or her boots,” show the other one up in public, tell the other he or she was going mad), and isolating control (6 items) (e.g., try to restrict time one spent with family or friends, want to know where the other went and who he or she spoke to when not together, try to limit the amount of activities outside the relationship the other engaged in). A meta-analysis of 98 studies spanning 2013 to 2023 across 40 countries utilized assessment instruments including the WHO Women’s Health and Life Experiences Questionnaire (WHO-WHLEQ), Abuse Assessment Screen (AAS), WHO Violence Against Women Instrument (VAWI), and Conflict Tactics Scale (CTS/CTS2), revealing IPV prevalence estimates between 9 and 26%: physical violence (10%), psychological violence (26%), sexual violence (9%), verbal violence (16%), and economic violence (13%) (Brunelli et al., 2025). The Conflict Tactics Scale continues to be the predominant standardized measurement tool for IPV, available in both self-administered and interview formats with robust psychometric properties. However, critics note its limitations in capturing injury severity, coercive control, self-defensive actions, and the contextual factors or motivations driving violent behavior (Costa & Barros, 2016). Differences in research design, population samples, how IPV is operationally defined, assessment timeframes, and chosen measurement tools all influence reported IPV disclosure rates (Brunelli et al., 2025). Together, these efforts informed our conceptualization of IPV and guided our study to detect the features and characteristics of IPV from social media websites.

### Qualitative Analysis and Text Mining of Blog Texts

Online media platforms are increasingly used by individuals to share their thoughts and experiences on specific topics of concern and interest (Subramani et al., 2017). Such online platforms provide an important avenue for researchers, professionals, and other stakeholders to gain insights into women’s lived experiences of IPV. Online forums dedicated to IPV provide safe, anonymous spaces for survivors to share their experiences, seek emotional and informational support, and connect with others facing similar challenges. These forums often function as peer-to-peer support networks, offering validation and coping resources when formal services are inaccessible or perceived as unsafe (Andalibi et al., 2016; Chu et al., 2021). Prior studies have shown that participation in such forums can foster empowerment, self-reflection, and help-seeking (Tarzia et al., 2018), though some research also highlights potential risks such as retraumatization or exposure to harmful advice (e.g., Chu et al., 2021).

Because of the volume, detail, and ecological validity of the posts shared in these spaces, forum texts represent a valuable but underutilized data source for IPV research. Text mining as a cutting-edge technology has been widely used in the field of information science and linguistic computational science over the past decade (Allahyari et al., 2017). Text mining includes the use of computational methodologies and tools to analyze the semantics, grammatical, linguistic, and syntactic patterns of text data. After text data is entered into the computer, the computer generates classification algorithms based on comparative unique-word frequencies and word combinations (Allahyari et al., 2017). These methods allow researchers to examine semantic, linguistic, and syntactic patterns across extensive narratives, revealing trends that may be difficult to detect through manual qualitative analysis alone. For IPV research, text mining is especially advantageous because it can process large volumes of survivor-generated content, capture nuanced experiences of abuse, and highlight contextual factors that may remain hidden in traditional research designs.

### Research Questions

This study was guided by three research questions. First, what range of IPV behaviors emerge through qualitative coding? Second, to what extent do ML-based text classifications yield results comparable to qualitative coding of IPV behaviors? Finally, do the conceptualizations that emerge from unsupervised ML capture additional behaviors or contextual information not identified through qualitative analyses?

## Methods

### Sample and Text Data Collection

For the current study, we extracted data from survivors’ postings about their experiences of IPV on three widely used independent online IPV aid forums: Women’s Aid Survivors’ Forum, WEAVE Domestic Violence Message Board, and Fortre fuge Forum. These forums were identified using Google searches with the keywords “domestic violence support forum” and “women share stories of survival.” We conducted a comprehensive search for IPV-related forums across English-speaking countries and retained the largest, most active platforms for analysis. These forum posts allow us to capture a broad range of survivor experiences and linguistic expressions of IPV. At the time of the internet search between 2000 and 2021, there were 4600 posts by survivors. We extracted posts that included 30 or more words. Short posts often lack contextual or semantic richness, which can reduce the reliability of both qualitative coding and machine learning classification (Teferra et al., 2024). We also only include posts where survivors used terms such as “my husband”, “my boyfriend”, “my partner”, “my ex” or terms such as “he”, “him”, or “his” to refer to their intimate partners were included in the analyses. This focus ensured a more homogeneous dataset suitable for text-based ML analyses, which perform best with large, thematically consistent text samples. Ensure sufficient linguistic content for meaningful analysis. The final data set included 400 posts from survivors who identified themselves as victims of violence. Approval for the study was granted by the institutional review board. Data were collected only from publicly accessible IPV forums whose content is visible without registration or membership. We have also taken extra precautions to anonymize (e.g., removal of forum usernames, locations, etc., and only extract narratives) and aggregate the data to prevent any potential identification of individuals, adhering to ethical research practices.

Survivors posted and viewed the social media posts of others without providing much private information. Only a few of the social media posts included sociodemographic and background information. One hundred eighty participants (from the 400 posts) indicated that they were married. Children were mentioned in 119 posts as being directly involved or having witnessed parental violence or as reasons for staying/ending the abusive relationship. Abusers’ histories of substance use and mental health issues were mentioned as potential reasons for the abuse (55 posts and 15 posts respectively). In 90 posts, survivors reported that they had taken legal actions against their perpetrators by calling the police, applying for a restraining order, and/or registering criminal complaints against the perpetrators.

### Analytic Strategy and Procedure

**Fig. 1:**
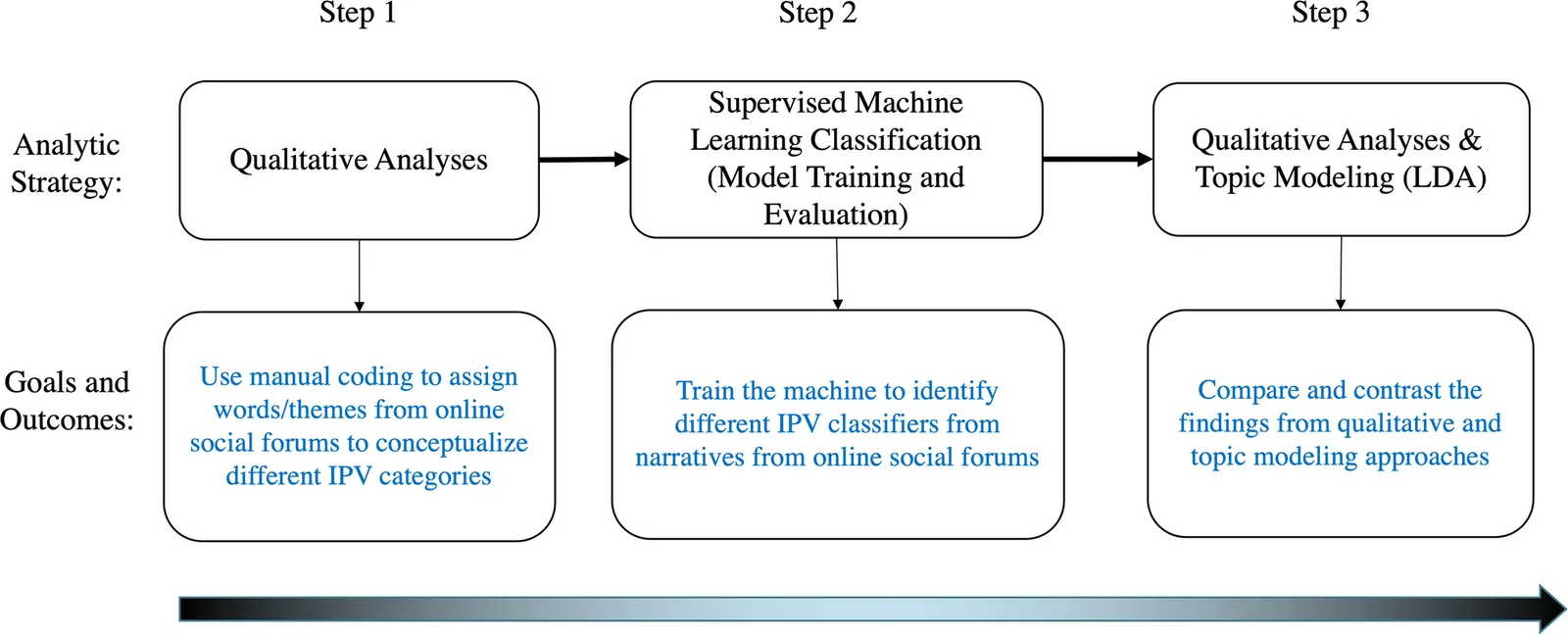
Flow chart of analytical strategy and results

The analytic strategies and results used in the qualitative (content analysis approach; Vaismoradi et al., 2013) and ML are organized under separate subheadings. The flow chart for the analytic strategies is indicated in Fig. 1.

#### Qualitative Analyses

After data cleaning (e.g., removing notes from the website coordinators) and de-identification of recognizable data, narratives were manually coded (in Microsoft Excel) based on the words and expressions of the victims (see codebook from Table 1). Narratives from each blog on the social media site were read by the first author who subsequently assigned succinct qualitative labels to different segments of texts. For example, “He hit me from my bedroom with fists all over my body and face” was coded as “physical violence” and “I’m being emotionally blackmailed into staying with him like he’s trying to wear me down until I say yeah let’s try again” was coded as psychological violence. For this project, we combined physical and/or sexual IPV behaviors by an intimate partner or non-partner or both because few instances of sexual violence were seen in women’s IPV narratives. A codebook with qualitative labels along with explanations for the labels (e.g., behaviors characteristic of the code) was developed first, guided by predefined categories derived from the IPV literature and measurements (see Table 1). To ensure data reliability, two doctoral students with expertise in the field of IPV independently coded 20% of the social media posts (*n* = 80). The two coders agreed on 94.09% of the codes. Discrepancies between coders about labeling the codes/labels were resolved through discussion.

**Table 1:** Codebook for qualitative analysis of social media texts

Constructs	Definition
Physical and sexual violence	Text indicating the victim’s experiences of physical force, such as hitting, beating, or kicking by the perpetrator. Text indicating the perpetrator’s use of force that compels the victim to involuntarily participate in a sex act and/or sexual touching or non-physical sexual activity (e.g., sexting) to which the victim does not or cannot consent
Psychological violence	Text referencing the perpetrator’s use of psychologically abusive tactics intended to demean the victim (e.g., calling the victim names, playing mind games, humiliating the victim, making the victim feel guilty)
Coercive controlling behaviors	
Coercion and threats	Text indicating the perpetrator’s use of verbal intimidatory tactics to threaten the victim (e.g., threatening to hurt the victim, leave the victim, commit suicide, report the victim to authorities, force the victim to drop charges to authorities, force the victim to conduct illegal acts)
Intimidation	Text referencing the perpetrator’s use of nonverbal actions to threaten the victim (e.g., looks, actions, and gestures), smashing and destroying property, abusing pets, and displaying weapons
Isolation	Text indicating the perpetrator’s control over what the victim does, whom the victim sees and talks to, and what the victim reads thereby limiting the victim’s life and activities
Minimizing, denying, and blaming	Text that signifies the minimization of the violence inflicted on the victim by the perpetrator. Text that references the denial of the violence and/or shifting the responsibility for violent behavior to the victim
Using children	Text referencing the perpetrator’s use of children to make the victim feel guilty, using children to demean the victim, and threatening to take the children away from the victim
Economic abuse	Text indicating the perpetrator’s actions that prevent the victim from getting or keeping a job, limiting, or not giving the victim access to money and resources
Male privilege	Text stating the perpetrator’s acts of superiority over the victim. Text indicating that the perpetrator is the “master of the castle” and references patriarchal notions of hierarchy in relationships
Stalking behaviors	Text referencing the victim’s experience of a pattern of repeated, unwanted, harassing actions (e.g., threatening phone calls, emails, covert surveillance) by the perpetrator to gain attention and contact with the victim

#### Supervised Machine Learning Classification

Following the qualitative analyses, we used ML approaches on the same online social media dataset to train the computer to learn word patterns (“supervised text classification process”). Supervised ML analysis used researcher-defined a priori categories derived from our qualitative coding. To ensure a balanced sample size in this violence-free reference category, we combined 84 posts from online forums that were free of violent content with paragraphs extracted from books that focused on romantic relationships among young adults. By merging these two distinct sources, we have a diverse corpus of non-violent text. In the data preprocessing step, text mining approaches were used to convert free (unstructured) text data into normalized, structured data using the Natural Language Toolkit (NLTK) (Allahyari et al., 2017). To train the computer to analyze the semantics, grammatical, linguistic, and syntactic patterns of text data (Allahyari et al., 2017), several steps (five) were undertaken. First, irrelevant items, such as punctuation and trivial words were removed and every letter in the text was converted to lowercase in a procedure termed “normalization” (Bird et al., 2009; Weiss et al., 2015). Second, in a process termed “tokenization”, all words in a sentence were converted into a sequence of words or tokens that had meaning. This step was undertaken up to the point where the word could not be meaningfully split up any further (Webster & Kit, 1992; Weiss et al., 2015). Third, words such as “the”, “a”, and “at” were removed from the text as they are considered “stop words” that are unlikely to contribute to the understanding or analysis of the text. Fourth, the remaining words were then converted into their dictionary form using two processes: stemming and lemmatization (Balakrishnan & Lloyd-Yemoh, 2014; Weiss et al., 2015). In the lemmatization procedure, words with similar meanings were converted to a common base form to improve the model’s ability to recognize related concepts. For example, different grammatical forms of a word (e.g., “threatens,” “threatened,” “threatening”) were standardized to their base form (“threaten”). This process reduces vocabulary complexity and allows the algorithm to detect patterns of threatening or coercive behavior regardless of tense or phrasing. Fifth, all words in the text were converted into a vector of scores via “Term Frequency, Inverse Document Frequency” (TF-IDF) which allowed us to assign frequencies to the usage of specific words within each document. Words received higher TF-IDF scores when they appeared frequently within one document and served as unique identifiers of a concept. On the other hand, words were given lower TF-IDF scores when they appeared frequently across several documents and hence could not be considered suitable identifiers (e.g., the “relationship” showed up frequently across documents and as such received a lower TF-IDF score).

**Fig. 2:**
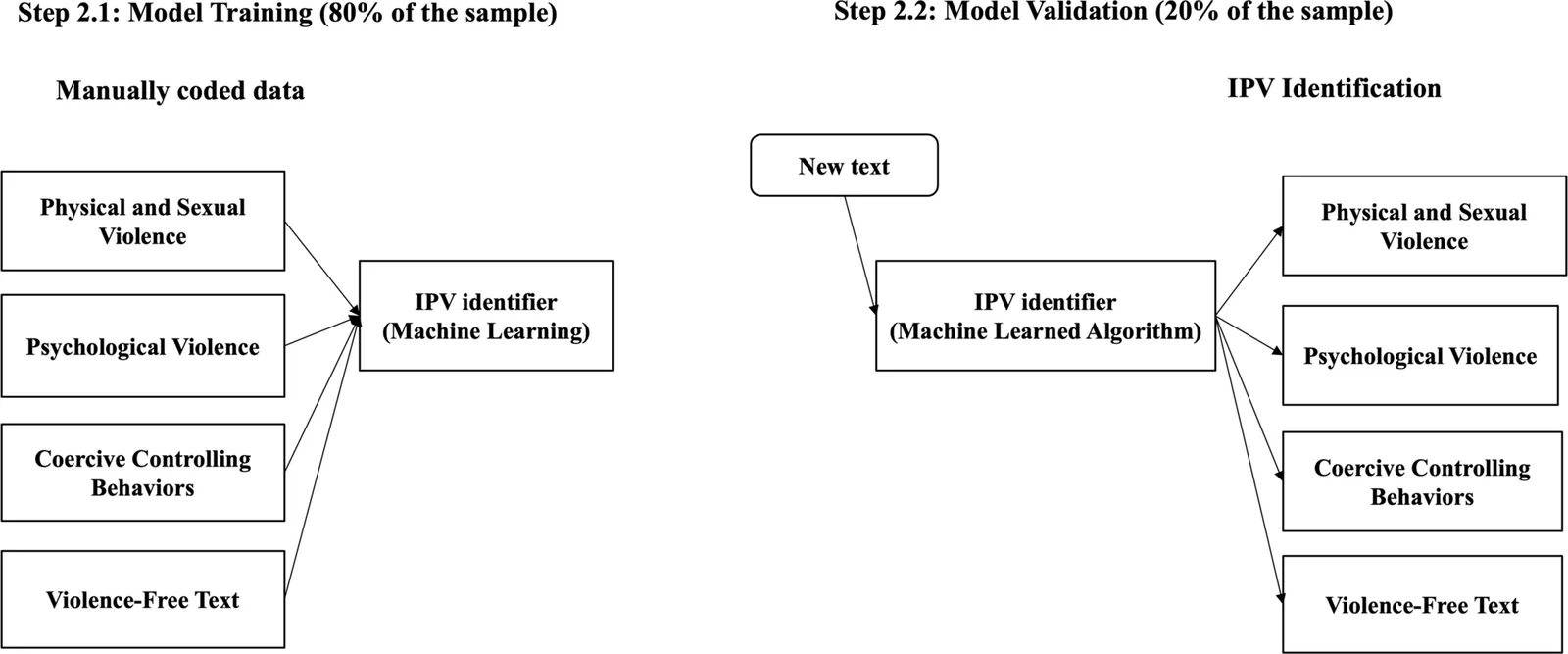
Classification training and model validation procedures

We used three ML algorithms, Random Forest (RF), Neural Networks (NN), and Supported Vector Machines (SVM) (Pedregosa et al., 2011; Weiss et al., 2015) to assemble different IPV classifications (i.e., grouping, and labeling words or phrases into categories such as physical violence, sexual violence, psychological violence, coercive controlling behaviors, and non-violence) and to ascertain the accuracy of each algorithm to predict different IPV categories. This step was undertaken with 80% of the data (Model Training) (see Step 2.1 in Fig. 2). The *RF* algorithm is an ensemble method that leverages the strength of multiple decision trees. By aggregating the predictions from each decision tree, *RF* effectively mitigates the risk of overfitting the model thereby enhancing its overall accuracy. The *NN* algorithm develops layers of interconnected nodes that are organized hierarchically, with the last layer producing the final label. This algorithm is capable of learning complex patterns and achieving high-accuracy results. Finally, the *SVM* algorithm is especially useful when handling high-dimensional and non-linearly separable data by transforming input data into a higher-dimensional space. The accuracy results are ascertained by leveraging the unique strengths of each algorithm. After we trained the machine in identifying different IPV categories, we validated the performance of the classifiers to assess how well they performed on new data (the remaining 20% of the sample). The classification accuracy of different ML algorithms (Model Validation) (see Step 2.2 in Fig. 2) was examined particularly in their ability to predict diverse IPV categories.

In ML classification, it is common for categories or labels in a dataset to show high correlations, reflecting the co-occurrence of different forms of behaviors. In our study, when labels such as “psychological violence” and “coercive control” are highly correlated or tend to co-occur, ML can detect and learn from these relationships. Additionally, we employed multi-label classification, enabling an instance to be categorized into multiple labels. For instance, an individual case could be classified as both psychological violence and coercive control. This methodology allows the model to effectively manage correlated categories without necessitating a binary decision between them. By supporting multi-label assignments, the model recognizes that these behaviors can occur simultaneously, thus maintaining the nuanced relationships among the different types of IPV.

Our examination of the IPV data before using supervised ML algorithms (Random Forest (RF), Neural Networks (NN), and Supported Vector Machines (SVM)) indicated data imbalance between IPV categories i.e., there were 254 posts on physical violence compared to 40 posts on sexual violence. Because supervised ML algorithms require balanced data and if the data is unbalanced, the machine algorithm will be biased toward the majority class (Batista et al., 2004). As such, it was necessary to merge the sexual violence category with the physical violence category. This approach resulted in four relatively balanced IPV categories, i.e., physical/sexual violence, psychological violence, coercive controlling behaviors, and violence-free text.

The classification accuracy of the IPV categories (Model Validation) was evaluated using indicators of precision (quantifies the model’s ability to make accurate positive predictions by avoiding false positive errors) and recall (known as sensitivity or true positive rate which focuses on minimizing false negatives) (Hastie et al., 2001; Weiss et al., 2015). Each ML algorithm assessed a class-specific *F*1 score (harmonic mean of precision and recall) for every IPV category. A high *F*1 score indicated a good balance between precision (accurately predicting positive instances) and recall (capturing all positive instances) (Sokolova & Lapalme, 2009; Weiss et al., 2015).

#### Unsupervised Topic Modeling

The unsupervised ML approach that we employed was the Latent Dirichlet Allocation (LDA) algorithm (a probabilistic generative model referred to as topic modeling) (Ramage et al., 2009; Vayansky & Kumar, 2020; Wallach, 2006). The algorithm can uncover words that reflect different IPV categories based on their observed patterns of occurrence. Unlike supervised machine learning, which requires labeled examples to train a classifier, topic modeling does not rely on predefined categories; instead, it infers latent semantic structures directly from patterns of word co-occurrence. The data preprocessing procedures (tokenization, lowercasing, stop-word removal, and stemming) were identical to those used in the supervised ML analyses to ensure consistency across methods. We conducted separate analyses for posts categorized as (a) physical and sexual violence, (b) psychological violence, and (c) coercive-control behaviors to identify topic patterns specific to each form of IPV.

## Results

### Qualitative Findings

Results of the qualitative analysis indicated the presence of multiple characteristics of IPV (see Table 2).

**Table 2:** The IPV categories and the corresponding number of narratives

IPV categories	Subcategories	Number of narratives
Physical and sexual violence	Physical and sexual violence	294
Psychological violence	Emotional abuse, verbal abuse	212
Coercive controlling behaviors	Male privilege, economic abuse; using children; minimizing, denying, and blaming; isolation; stalking; coercion and threats; intimidation	259
Violence-free text^a^	No violence	200

^a^To ensure a balanced sample size in this violence-free reference category, we included 84 posts from online forums that were free of violent content with paragraphs extracted from book chapters that focused on romantic relationships among young adults

#### Physical and Sexual Violence

Victims’ experiences of physical violence were indicated in 254 out of the 400 posts with reports of being hit, beaten, and/or kicked by their perpetrators. Physical violence occurred in residential settings and/or inside cars. In a few cases, weapons such as knives or shotguns were used. Victims indicated their experiences of sexual violence in 40 posts with words such as sexual assault, abuse, force, and rape to describe their experiences. Below is one example of one such post.

My ex-boyfriend has severe anger problems that seem to be getting worse as time goes by. In the past week it reached new levels that I have never seen before. He grabbed me, swung a glass bottle towards the back of my head (it did not hit me), and threw a chair across our living room.

#### Psychological Violence

Victims reported their experiences of psychological violence in 212 posts stating that their partners used mean and derogatory words to put them down and make them feel bad, useless, incapable, and humiliated. Some perpetrators made cruel and insensitive “jokes” in the victim’s presence.

….constantly nags for hours and when he goes outside for cigarettes he nags and complains about me or kids to himself (full-blown conversation). He will sit there and call me names talking to himself he thinks he’s not crazy…Constantly calls me fat says kill myself talks about my past and my first husband that died like he knows him or my past.

#### Coercive Controlling Behaviors

*Coercion and threats*. Victims reported that their partners used various coercive and threatening behaviors (170 posts) such as threats of self-harm, economic threats (not working or not contributing financially to the family), emotional withdrawal, disclosure of victims’ illegal status, and disclosure of victims’ past “inappropriate” actions. In 95 posts, coercive threats included the use of physical violence. Even without physical harm, survivors who reported coercion and threats stated that they felt controlled, suffocated, and scared to leave their abusive relationships.

He asked me if I still wanted to be with him and I said no. He then said twice that I’m going to make him commit suicide. I wanted to get out of the car and go inside my house but he started to drive and he asked me to punch him. I of course didn’t and then he punched himself twice in the face.

*Intimidation.* In 97 posts, victims stated they felt afraid and scared by their partner’s looks, actions, and gestures. Abusers intimidated their partners by threatening to use weapons and hurt family members and/or pets.

I was forced to move twice because he kept finding me and we faught (fought) so often and he would run before anyone could call the police. There were a lot of damages to the apartments and I was forced to move with not enough money to take everything or the safety to even go back to the apartment being that I lived alone with my 3-year-old son. So, both times we were forced to move to shelters and start over.

*Isolation*. In 97 posts, victims reported that they were sequestered from their families and friends by perpetrators.

…has somehow isolated me from the world. I use to know… Is it ok to still maybe get help or counseling from somewhere? I’ve become financially and in all ways codependent on him. He wouldn’t chase me if I left he’d just let me go.... but I don’t know where to go.

*Minimizing, denying, and blaming*. Victims reported that their partners used alcohol and illegal substances as an excuse for their use of violence (78 posts). Perpetrators minimized their use of emotionally and verbally abusive language by claiming that the words were said in jest and not intended to be taken seriously. In some instances, male perpetrators shifted the blame for the violence to their victims.

And he start be mean to me, insulting, humiliating, blaming me for anything. He left me in a strange and dangerous place without phone or car. He start says our marriage was a terrible mistake, and then he avoided me completely, days and days without talk to me. He can be angry for anything, and scary yelling and driving very fast on propose. He touches my intimate parts when I am sleeping, I already wake on middle of night when he did that. He said everything of bad in his life is my fault because we are married….

*Economic abuse*. In 43 posts, victims reported that the perpetrators restricted their access to financial resources. Perpetrators prevented victims from having personal bank accounts, accessing money, or going to work. In some cases, abusers confiscated and took away the victim’s possessions.

I am always the blame for his anger, the finances, his debt, it never stops, he controls the shopping, the laundry soap, tells me to slow down on the cream, the bread whatever. I feel like a nobody and am craving to get out of here, he threatens to sell the house, go into foreclosure on a regular basis and breaks things I adore. He goes through my things and after 10 years I am not allowed a mailbox key.

*Using children*. In 173 posts, victims indicated that children had witnessed the perpetrators’ use of physical and/or psychological violence. In some instances, perpetrators used threats to harm, take away the children, and/or even threaten to take away the victims’ custody rights.

My partner and I have a 2 year old son together a house together have been together for 3 years and in that time he’s beat me down so much calling me a liar and a cheat saying our son wasn’t his I even paid for a private DNA test to prove otherwise it’s only when he gets really drunk he’s like this but it’s extreme he has stopped saying poor sons not his cause he know he is his but we had a fight 3months ago and kicked me and my 2 year old out at 2am he was very aggressive at this point but he spent a few days drinking sending a few threats about custody and then backed down saying we’d discuss it see what suited me.

*Male privilege*. In 18 posts, victims reported that their partners insisted that they comply with traditional gender roles and that the victim had to acquiesce to the will of the abuser. Some perpetrators used male privilege along with psychological violence to control their partners.

When he was confronted with the fact that he had 2GB of rape and bondage porn on his computer and having an affinity for teenagers (all in open court) he admitted to liking to control his partners and to keeping me on an ‘allowance.

*Stalking*. In 27 posts, victims reported on their partners’ stalking behaviors. Perpetrators engaged in a pattern of repeated, harassing actions to gain attention and contact with the victim. Some posts mentioned that children were used by the perpetrator to gain access to the victim. Fear and threats were also used to control the victim.

I could have and would have never gone to a home as my husband would have followed me or come to my place of business and waited and grabbed me and then taken me somewhere and beat me or killed me.

In the 259 posts, one or multiple coercive controlling behaviors were indicated.

#### ML Classification Results

As shown in Table 3, ML algorithms vary in their ability to predict diverse IPV categories. Based on information from the *F*1 scores, the *NN* and *SVM* algorithms had the highest *F*1 scores when predicting instances of physical/sexual violence. On the other hand, the *RF* algorithm exhibited better performance when predicting psychological violence and coercive controlling behaviors. The *NN* algorithm showed the highest *F*1 scores when detecting violence-free text, implying its proficiency in correctly classifying situations that did not involve any form of violence.

**Table 3:** The classification results for each IPV category

IPV	Precision			Recall			*F*1 score		
	*RF*	*NN*	*SVM*	*RF*	*NN*	*SVM*	*RF*	*NN*	*SVM*
Physical and sexual violence	0.63	0.72	0.69	0.87	0.79	0.83	0.73	0.75	0.75
Psychological violence	0.77	0.60	0.74	0.59	0.71	0.54	0.67	0.65	0.62
Coercive controlling behaviors	0.77	0.63	0.63	0.76	0.79	0.85	0.77	0.70	0.72
Violence-free text	0.95	0.95	0.97	0.72	0.76	0.70	0.82	0.85	0.81

Notes.* RF* = random forests, *NN* = neural network, *SVM* = support vector machine

#### Topic Modeling Results

Findings indicated that words such as hitting, slapping, or choking were representative of physical violence victimization. Words such as “sexual”, “rape”, and “force” were representative of sexual violence. Similarly, words such as blame, threat, and accusations were representative of psychological violence. Words that included tactics such as isolation, intimidation, limiting access to money, isolating the partner from friends and family, and employing threats and intimidation were characteristic of coercive controlling behaviors. We conducted a manual inspection based on the top words associated with each IPV representation and checked how well they reflected each theme (e.g., “hitting” and “slapping” for physical violence). Findings indicated that the grouping of the words under different IPV categories was appropriate.

#### Comparison of Findings from Qualitative Analysis and Unsupervised ML

A comparison of the findings from the qualitative analysis and the topic modeling approaches indicated significant similarities (see Table 4). Findings also pointed to additional information gained using the topic modeling approach. Some words that were extracted provided valuable insights into the emotional, relational, temporal, legal, and spatial dimensions of IPV. These findings supplemented the information gained using qualitative approaches. For instance, topic modeling picked up words like “threat,” “anger,” “anxiety,” “felt,” “worried,” “guilty,” “wonder,” “safe,” “pain,” “apart,” “fault,” “upset,” “terrified,” and “horrible” that described emotions associated with IPV. The topic modeling algorithm also picked up words related to children, such as “custody,” “school,” “visit,” “parent,” “pregnant,” and “apart”; words related to the timing and frequency of violence, such as “continue,” “constant,” “recent,” and “begin”; words associated with the use of legal resources, including terms like “officer,” “lawyer,” “legal,” “plan,” “drop,” “state,” “counsel,” “cop,” “prosecute,” “court,” “divorce,” “jail,” and “advice”; and words related to the location or context of violence, such as “room,” “door,” “floor,” “place,” and “office.”

**Table 4:** Comparisons of words in each violence category as identified by qualitative analysis and topic modeling

Constructs	IPV words (qualitative analysis)	IPV words (topic modeling)
Physical and sexual violence	Hit, physical, punch, bit/bite, beat, kick, push, shove, strangle, choke, sex/sexual abuse, sexual assault, sexual force, rape	Hit, choke, force, slap, victim, assault, argument, fall, scream, aggressive, sexual, hair, pull, yell, attack, sexual, rape, physic, hurt, force, violence, break
Psychological violence	Emotional abuse/abusive guilty (made victims feel)	Blame, threat, anxiety, argument, confuse, fault, spend, heart, apart, stupid, accus, concern, guess, worry, chance, deal, force, confuse, upset, restrain, treat, anger, accus, nice, mind, kind
Coercive controlling behaviors	Threat/threaten, blackmail, scared/scary, fear, destroy (things), smash, (presenting) gun, (presenting) weapon (abuse/kill) dog/cat, isolate, jealous(y), keep (at home), blame, guilt(y), deny, minimize, (denied) bank account, income/money, stalk, harass(ment), follow, hack, make child against her, (need) approval/permission, rules, “old fashioned”	Normal, concern, scream, argument, continue, manage, worry, constant, fault, bear, finance, apart, stand, upset, guilty, confuse, wonder, message, cry, fear, apart, sorry, differ, apology, end, guess, buy, scary, threat, parent, social, restrain, stupid, couple, afraid, pay, deal file, heart, kind, drug, visit, lie, medical, attack, shout, safe, allow, incidence, accuse

## Discussion

Violence against women is a significant public health concern and remains one of the most serious barriers to women’s well-being and full participation in society (UN Women, 2025). Understanding how survivors describe their experiences, needs, and challenges is essential for improving support and services for individuals and families affected by IPV. In recent years, researchers have begun to examine new sources of IPV-related data, such as publicly available posts on social media to better understand how survivors articulate their experiences in digital spaces (Subramani et al., 2017). These sources can complement traditional qualitative and survey-based approaches by providing additional insight into the language, contexts, and themes that emerge when survivors share their stories online. Our findings are descriptive in nature and are aimed to deepen understanding of survivors’ online IPV narratives. Our overall observation suggests that integrating ML with qualitative coding provides an additional lens for understanding the language and contextual dynamics embedded in survivors’ narratives. The findings suggest that both supervised and unsupervised ML are feasible and valuable methodological tools for coding and analyzing large-scale textual data. When used responsibly, ML can complement and enhance manual qualitative approaches by identifying patterns that may not be readily visible through human coding alone.

Using supervised ML, we were able to train the computer to generate algorithms that identified four types of IPV: *physical/sexual violence*, *psychological violence*, *coercive controlling behaviors*, and *no violence*. Words such as hit, choked, force, slapped, sexual, rape to signify survivors’ experiences of physical/sexual violence. Victims indicated that perpetrators used mean and derogatory words to “put them down” and these words (calling victim names, gaslighting, and/or humiliating) were grouped to indicate psychological violence. *Coercive controlling behaviors* included victims’ use of words such as threat/threaten, blackmail, scared/scary, fear, destroy things. Our findings present how ML can enhance our understanding of various abusive behaviors of controlling behaviors expressed in online posts, including perpetrators threatening self-harm, threatening to disclose information about illegal behaviors, threatening to use/hide drugs as a means of exerting control, and threatening infidelity or disloyalty. These findings demonstrate that employing automated ML tools can capture subtle IPV dynamics, based on the nature and characteristics of survivors’ language expressions that existing IPV surveys fail to capture. These advances can certainly provide insights into clinician training on IPV-related issues. Suggestions include the development of screening tools and assessment protocols informed by survivors’ language patterns and clinical guidelines based on the range of IPV behaviors revealed through these study findings.

Findings indicated that survivors experienced multiple forms of violence in their relationships like findings from the substantial research on IPV (Graham-Kevan & Archer, 2005; Tiwari et al., 2015; Walker et al., 2021). In terms of the performance of the three ML algorithms, *NN* and *SVM* algorithms achieved the highest accuracy in predicting physical/sexual violence instances. Meanwhile, the *RF* algorithm excelled in identifying psychological violence and coercive controlling behaviors. These findings suggest that ML algorithms differ in their accuracy (although not very large) when predicting different IPV categories. In the unsupervised ML, topic modeling analysis operated independently of our broad, established IPV categories (physical/sexual violence, psychological/emotional abuse, and coercive control), and uncovered additional layers of contextual detail that human coders might overlook, such as the emotional, relational, temporal, legal, and spatial dimensions of IPV experiences.

### Strengths and Implications

This study has several strengths. Our study, while only accessing a fraction of the available large social media database on IPV, was able to demonstrate the effectiveness of the automated analysis method (LDA) to identify common themes and unique features of IPV. ML approaches could help researchers to code large volumes of data at a faster pace thereby furthering our understanding of how survivors understand and cope with their IPV experiences. While the research presented in this study is among a limited number of studies employing ML and social media data analysis to comprehend IPV manifestations, it holds significant potential. Our findings may be instrumental in the future development of effective online tools for first responders who handle domestic violence cases, aiding in better identification and support for victims. The limited availability of objective IPV detection tools can undermine police officers’ decision-making when attending to IPV-related distress calls. As such, ML algorithms can be helpful in the development of Apps on cell phones and websites which can be incorporated into the technological devices carried by police officers.

This research contributes to expanding the knowledge base in the field and highlights the importance of leveraging ML techniques for enhancing intervention and support systems related to IPV. For example, the further developed tool helps provide feedback to assist survivors with decision-making and/or to direct victims to the IPV service providers (Zhang et al., 2025). Algorithms, such as those generated from this project could be used to develop an interactive website and independent Apps for mobile phones. These technologies can help victims understand the nature of violence that they are experiencing in their relationships as well as assist in their decision-making. Specifically, this study lays the groundwork for creating an interactive website designed to help survivors gain an early understanding of abusive relationships. By rapidly analyzing patterns of IPV, this tool can offer survivors real-time insights into their situations, potentially helping them recognize signs of abuse earlier (Zhang et al., 2025). For example, we envision an interactive platform that can analyze patterns of IPV in real time, offering survivors early insights into abusive behaviors. By providing personalized, data-driven feedback, such a tool can help survivors recognize signs of abuse sooner and seek timely support. The IPV-related phrases and language used by survivors on social media sites can be informative in the development of truly survivor-centered treatment programs. It is noted that minority women survivors are less likely to divulge their experiences to formal service providers (e.g., law enforcement, and healthcare providers) and more likely to report their experiences on social media and to friends and family.

ML algorithms can be efficiently used to analyze large volumes of text data, enabling researchers to speedily identify patterns and trends related to IPV in a timely fashion. The use of unsupervised ML (i.e., topic modeling) analyzed the text separately on broadly defined IPV categories (physical/sexual violence, psychological/emotional abuse, and coercive control), revealing patterns that might not be immediately clear to human coders (such as insights into the emotional, relational, temporal, legal, and spatial aspects of IPV). This approach, in contrast to simple keyword searches, extends beyond mere frequency counts. It leverages advanced natural language processing techniques to account for the nuanced ways in which words combine to convey meaning. This includes understanding synonyms and contextual usage, which are critical for accurately interpreting sentiments or thematic content in textual data (Momtazi & Naumann, 2013). Collecting IPV data from multiple and varied sources, such as electronic health records, surveys, and social media platforms, can enhance the creation of precise ML algorithms that advance both IPV research and real-world applications (Hui et al., 2023). Including contextual information about IPV circumstances and risk factors can support the development of sophisticated and more responsive algorithms capable of providing early detection signals for IPV (Cruz-Mendoza et al., 2025). Equipping researchers and counselors with ML algorithm training can facilitate the identification of individuals at elevated violence risk, enabling prompt intervention that surpasses traditional methods (Cruz-Mendoza et al., 2025). Following identification, counselors can provide customized treatment, support, and services to IPV survivors that are specifically designed to meet their unique circumstances and needs (Hui et al., 2023).

Given that many IPV victims are reluctant to respond to sensitive questions about their experiences of IPV, the use of victims’ self-reports (with closed-ended questions) by shelter agency staff may not be very informative. For many IPV victims, this process seems more like “questioning” than a step toward resolving their problems (White et al., 2019). As such, victims are likely to withhold information to “protect” the abuser and the family. If survivors are allowed to safely narrate their stories to shelter staff and the qualitative data is made available to ML apps, this analysis of information can better serve IPV victims. In developing technological solutions for supporting IPV survivors, it is also crucial to adopt trauma-informed practices that prioritize the safety and experiences of survivors. This approach emphasizes the development of features and safeguards that are sensitive to the psychological and emotional state of individuals who have experienced trauma. Therefore, with many promises that ML presented in this study, we view ML-enabled algorithms as a supportive tool rather than a replacement for trained professionals in handling complex and sensitive situations involving IPV.

### Limitations and Future Directions

This study is not without its limitations. Although we used three estimation methods (*NN*, *RF*, and *SVM* algorithms) because of their simplicity, quick convergence, and versatility in model building (Weiss et al., 2015), it should be noted that there are other algorithms such as *deep neural networks* that could also be used to determine accuracy in classification. Future studies should include additional algorithms to assess IPV classification accuracy. One of the challenges in advancing classification accuracy is the co-occurrence of various types of IPV in relationships (e.g., physical violence and psychological violence). This complexity can lead to a reduction in classification accuracy, as the classifier needs to accurately predict multiple labels for each instance. For example, in a classification, the labels “psychological violence” and “coercive control” may be highly correlated and occur together. If the classifier predicts one of the labels, it is also likely to accurately predict the other label. To enhance the prediction accuracy, one possible solution is to include more cases to optimize the algorithm (Banko & Brill, 2001; Hossain et al., 2021).

We recognize the importance of aligning our categories and definitions with those set out by organizations such as the CDC. Our conceptualizations of the violence constructs (sexual violence, physical violence, stalking, and psychological aggression) do align with these definitions. However, our decision to identify coercive control as a separate construct is based on its legal recognition in the UK and the USA as a form of domestic abuse. Coercive control behaviors encompass force, threats, and control behaviors as well as actions used by perpetrators to isolate, manipulate, and coerce compliance from their partners. According to Johnson and others (Johnson, 2008, 2011), violence is added to a pattern of power and control in relationships and is the basis for various intimate partner violence typologies (e.g., intimate terrorism). Johnson notes that coercive control behaviors can occur in relationships without violent behaviors (i.e., nonviolent coercive control) (Johnson, 2008, 2011). Together, these theoretical and legal perspectives underscore the necessity of treating coercive control as a stand-alone category in IPV research, emphasizing its unique and pervasive nature. We encountered a challenge in the data analyses that we had to combine physical and sexual violence in our analyses. This decision to combine them was made because there were limited reports of sexual violence in the narratives that were part of the current study. Future researchers should analyze IPV narratives from larger and multiple social media sites. In doing so, algorithms can be developed to clearly distinguish between physical and sexual violence behaviors.

Several participants described their IPV experiences on social media websites without providing their demographic information. This limited the demographic information that could be garnered from participant postings. We decided to focus on posts written by female survivors. Although women experience IPV at disproportionately high rates, this focus restricts the generalizability of our findings. Male survivors, nonbinary individuals, LGBTQ + partners, and others with marginalized identities may describe violence differently, use distinct language patterns, or face unique forms of coercion and stigma that are not represented in this dataset. As a result, the linguistic patterns identified here primarily reflect women’s experiences and may not capture the full diversity of IPV narratives across gender groups. Future research should incorporate more diverse samples to better understand how IPV is experienced and articulated across different populations. Additionally, participants in the current dataset could include only those individuals who have access to the Internet and are willing to discuss their experiences in public or semi-public forums. As such, data included in this paper may not be representative of certain sociodemographic groups. Consequently, our analysis is better suited for identifying broader patterns and trends in IPV discussions rather than for individual identification.

Our ML models were trained and validated using a relatively large dataset drawn from three social media platforms. As such, our dataset captured the linguistic variability of IPV experiences as indicated by the narratives from our participants. However, over time new language and vocabulary around IPV experiences are likely to emerge and this is likely to be reflected in the usage of certain words and phrases. Hence, we propose that researchers should periodically retrain and validate different ML models with more recently collected data. This practice will ensure that the models are time-relevant and incorporate newer linguistic trends and usage patterns (Zanella-Béguelin et al., 2020). Researchers could also incorporate a Dynamic Updating Mechanism into the model’s architecture to continually update the model’s vocabulary and integrate different weights to different responses. The updating process can be semi-automated, combining automatic detection of potential new terms along with expert validation to maintain accuracy and relevance (Kumar et al., 2016). Recent studies have leveraged advanced methodologies, natural language processing (NLP), and qualitative analysis to examine IPV-related discourse on social media platforms, notably Twitter and Reddit (Kim et al., 2023). For instance, one study utilized qualitative content analysis to discern the characteristics and needs of IPV survivors discussed within IPV-related subreddits during the COVID-19 pandemic. Similarly, Al-Garadi et al. developed a natural language model designed to automatically identify IPV reports from Twitter, showcasing how ML and NLP can provide scalable solutions for monitoring and potentially intervening in real-time IPV occurrences (Al-Garadi et al., 2022).

## Conclusions

This project used text mining technology to extract word patterns from victims’ narratives of IPV on forum/social media websites. Our findings confirm the appropriateness of using ML approaches to assess the nature and characteristics of IPV using online social media data. The consistency of findings across the manual qualitative analyses and the unsupervised ML approach (i.e., topic modeling) indicated a high degree of similarity. This study contributes to prevention science by demonstrating how AI-driven approaches can enhance the detection and classification of IPV disclosures on social media. By streamlining data processing and enabling rapid analysis, ML techniques offer a scalable tool for early identification, risk assessment, and violence prevention in IPV cases. Our findings highlight the potential of AI to complement traditional research methods, ultimately improving the responsiveness of IPV prevention efforts and informing targeted support strategies for survivors.

## Data Availability

The data supporting the findings of our manuscript have been deposited in the Open Science Framework (OSF; https://osf.io/3abtj/).
